# Evaluating the peak-to-valley dose ratio of synchrotron microbeams using PRESAGE fluorescence

**DOI:** 10.1107/S0909049512005237

**Published:** 2012-03-15

**Authors:** N. Annabell, N. Yagi, K. Umetani, C. Wong, M. Geso

**Affiliations:** a RMIT University, Melbourne, Australia; b JASRI/SPring-8, Hyogo 679-5198, Japan; c William Buckland Radiotherapy Centre, Alfred Hospital, Melbourne, Australia; d School of Medical Science, RMIT University, Plenty Road, Melbourne, Victoria 3083, Australia

**Keywords:** PRESAGE fluorescent dosimetry

## Abstract

Synchrotron-generated microbeam radiotherapy holds great promise for future treatment, but the high dose gradients present conventional dosimetry with a challenge. Measuring the important peak-to-valley dose ratio (PVDR) of a microbeam-collimated synchrotron source requires both a dosimeter and an analysis method capable of exceptional spatial resolution. The PVDR is of great interest since it is the limiting factor for potential application of the microbeam radiation therapy technique clinically for its tissue-sparing properties (*i.e.* the valley dose should be below the tolerance of normal tissue). In this work a new method of measuring the dose response of PRESAGE dosimeters is introduced using the fluorescence from a 638 nm laser on a confocal laser-scanning microscope. This fluorescent microscopy method produces dosimetry data at a pixel size as low as 78 nm, giving a much better spatial resolution than optical computed tomography, which is normally used for scanning PRESAGE dosimeters. Using this technique the PVDR of the BL28B2 microbeam at the SPring-8 synchrotron in Japan is estimated to be approximately 52:1 at a depth of 2.5 mm. The PVDR was also estimated with EBT2 GAFchromic films as 30.5:1 at the surface in order to compare the PRESAGE fluorescent results with a more established dosimetry system. This estimation is in good agreement with previously measured ratios using other dosimeters and Monte Carlo simulations. This means that it is possible to use PRESAGE dosimeters with confocal microscopy for the determination of PVDR.

## Abstract 2

The peak-to-valley dose ratio of a microbeam array can be measured by fluorescence of PRESAGE dosimeters. Peak-to-valley dose ratios are calculated using this new technique and also by EBT2 film.

## 1. Introduction   

Microbeam X-ray irradiation offers an innovative and promising modality for delivering high dose to well defined sections of tumour-bearing tissue whilst sparing the adjacent healthy tissue. This approach relies on the superior regenerative ability of healthy cells compared with tumourous cells when repopulating a depleted region (Dilmanian *et al.*, 2007 ▶). Microbeam radiation therapy (MRT) requires a minimally divergent radiation source that passes through a collimator. For this purpose a synchrotron is used to generate highly collimated radiation. Such beams have been shown to have potential for the treatment of brain tumours that have high radiation resistance (Dilmanian *et al.*, 2007 ▶). MRT will soon be used in clinical trials at the ESRF facility, and offers significant hope for treating tumours that could not be treated with conventional radiotherapy (Martinez-Rovira *et al.*, 2010 ▶). In order to assess the potential clinical usefulness of a MRT beamline, the peak-to-valley dose ratio (PVDR) of the beamline must be thoroughly investigated, in order to ensure that a lethal dose is being delivered to the peak region but a sub-lethal dose is being delivered to the valley region. Numerous Monte Carlo studies have been performed to compute the dose profile of these beams (De Felici *et al.*, 2005 ▶; Nettelbeck *et al.*, 2009 ▶; Siegbahn *et al.*, 2006 ▶; Spiga *et al.*, 2007 ▶), but experimental verification of the dosimetry is highly challenging. To this end, the microbeam’s high dose gradient demands a dosimetry system that can faithfully resolve to micrometre-level precision. Also, a three-dimensional dosimeter should be used in order to validate the lack of beam diffusion.

There are now several options to choose from which to fulfil the requirement for high-resolution three-dimensional dosimetry, including Fricke gels and polyacrylamide gels. These dosimeters are limited by their requirement of a container, which makes them unable to resolve surface dose since the container will be unreactive. In contrast, PRESAGE dosimeters are solid plastic based and therefore do not require any container, thus making them an attractive option for creating a fully defined three-dimensional model of absorbed dose, including surface dose. PRESAGE is normally scanned using optical computed tomography (OCT) equipment such as the OCTOPUS system (Guo *et al.*, 2006 ▶; Sakhalkar *et al.*, 2009 ▶). This system uses computed tomography reconstruction of transmitted light images to produce a three-dimensional opacity distribution, which is a function of absorbed dose. The OCTOPUS system uses a laser of wavelength 633 nm, as this wavelength has been experimentally determined as having the peak visible absorbance for the oxidized version of leucomalachite green, the radiation-sensitive ingredient in PRESAGE (Adamovics & Maryanski, 2006 ▶). Recent work by Rahman *et al.* (2010 ▶) makes use of microscopic techniques in order to improve the spatial resolution of the OCT system, down to a spatial resolution of 25 µm.

Some of the advantages of PRESAGE dosimeters over other three-dimensional gel dosimeters are its low ion diffusion, approximate tissue equivalence, longer shelf life and the uniform response to differing energies and dose rates. Because of the solid polyurethane matrix, it has also shown to withstand periods of heat (Adamovics & Maryanski, 2006 ▶). The low ion diffusion of the polyurethane base makes PRESAGE an attractive option for measuring high dose gradients, such as collimated synchrotron microbeams, which requires dosimetry to be accurate to smaller than a cell in order to capture the biological effects of MRT. These advantages of the PRESAGE system have recently been utilized for the commissioning of a radiosurgery field less than 1 cm wide (Clift *et al.*, 2010 ▶). PRESAGE dosimetry has also recently been used to measure the dose delivered by the beam produced at the European Synchrotron Radiation Facility using computed tomography reconstruction of microscopic transmission data to achieve a resolution of 2.7 µm (Doran *et al.*, 2010 ▶). This work illustrated the capabilities of PRESAGE to accurately record a very high resolution dose image, but the technique used still required a light source to traverse the entire width of the rod, which introduces alignment uncertainty when measuring a MRT-collimated sample.

Although optically stimulated luminescence is an established form of dosimetry using Al_2_O_3_-based compounds (Yukihara *et al.*, 2008 ▶), PRESAGE has not yet been used in this manner. In this study the PVDR of the microbeams produced with the BL28B2 spectrum and set-up will be evaluated with PRESAGE and GAFchromic films. The GAFchromic film measurements taken in this work allow for comparison with previous work conducted on the same beamline by Crosbie *et al.* (2008 ▶), who used such films for the estimation of PVDR. This collimator produces microbeams with a nominal width of 25 µm separated by a gap of 175 µm (200 µm centre-to-centre). The PRESAGE gel was then analysed using the fluorescent channels of a confocal microscope, and compared with the results obtained on GAFchromic films.

## 2. Materials and methods   

### 2.1. PRESAGE dosimeters   

The PRESAGE rods used (20 mm diameter × 125 mm long) were purchased from Heuris Pharma, Skillman, NJ, USA, made using the method outlined by Adamovics & Maryanski (2006 ▶).

### 2.2. Radiochromic films   

The peak-to-valley ratio was determined using GAF­chromic EBT2, purchased from International Specialty Products (ISP Technologies). EBT films were selected to provide a comparison with an established PVDR measurement technique as used by Crosbie *et al.* (2008 ▶).

### 2.3. Irradiation   

**Figure 1:**
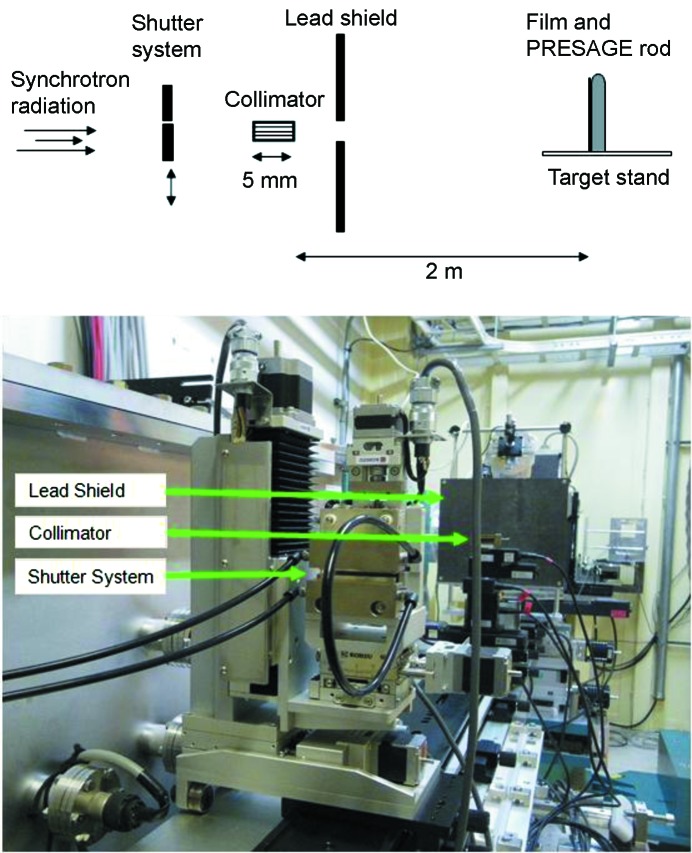
Top: schematic of the irradiation set-up at BL28B2. Bottom: photograph illustrating the set-up.

Samples of PRESAGE rods and EBT2 films were irradiated at the SPring-8 synchrotron facility in Harima, Japan, on beamline BL28B2 in November 2009. A collimator made from 5 mm × 20 mm plates of tungsten (175 µm thick) and kapton (25 µm thick) was used; it produced 25 µm-high beams with a gap of 175 µm, with the total beam pattern size being 1 mm high × 20 mm wide. Fig. 1 ▶ shows the set-up for irradiation. A 3 mm Cu filter was used, giving a peak fluence energy of 90 keV. The peak dose rate (air kerma) in the peak region was assumed as 120 Gy s^−1^, and the half-value layer was assumed to be 2.2 mm Cu, as previously calculated (Nariyama *et al.*, 2009 ▶). The spectrum of energy used in this work is specified in Fig. 1 of Nariyama *et al.* (2009 ▶), as calculated by *SPECTRA* codes. Dose was calculated as a function of exposure time using the shutter system, where the width of a shutter gap is set and the shutter is then moved across the beam at a well controlled speed, to produce a known exposure time. The collimator was located ∼40 m downstream from the source; beam divergence is thought to be negligible. The collimator set-up allowed for the irradiation of four microbeams at a time, for a total height of ∼1 mm, after which the target stand was moved to allow further microbeams. A total of eight peaks and seven valleys were made at each dose point for both films and PRESAGE rods. The peak dose rate (air kerma) was assumed to be 120 Gy s^−1^ (Nariyama *et al.*, 2009 ▶), and the dose was calculated as a function of exposure time using the shutter system.

**Figure 2:**
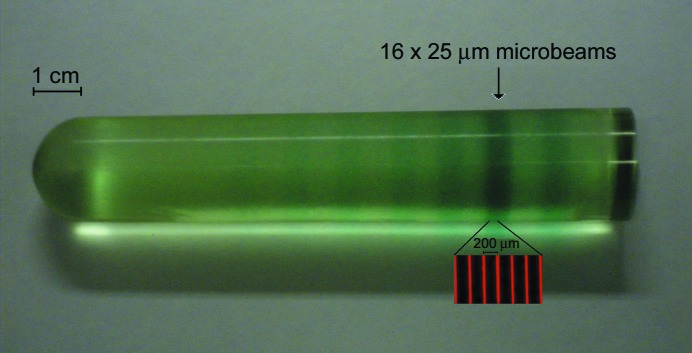
One of the rods of the PRESAGE used for this study, irradiated with different intensities of microbeams. The arrow points to an array of 16 microbeams (each 25 µm wide), difficult to distinguish with the human eye. Insert: fluorescence of a 638 nm laser on the PRESAGE dosimeter, under 10× objective magnification.

Fig. 2 ▶ illustrates a PRESAGE rod, highlighting the section that was irradiated at SPring-8. The insert of this figure shows the fluorescent scan of that section from the confocal laser scanning microscope (CLSM). For the analysis of beam diffusion at different depths of the PRESAGE, another rod was irradiated lengthwise instead of from the side. After irradiation, all rods were stored in a refrigerator (277 K) protected from UV light for approximately one month before being read with fluorescent microscopy.

### 2.4. Fluorescent microscopy for irradiated PRESAGE dosimetry   

A Nikon Eclipse Ti-E A1 CLSM was used to scan the PRESAGE dosimeters (Nikon Instruments, USA), using 10× and 40× objectives. Images were captured using the *NIS-Element* imaging software (version 3.2). The microscope is equipped with four lasers of wavelengths 405 nm, 488 nm, 561 nm, 638 nm. It also has four detectors with sensitivity spectra of 662–737 nm, 570–620 nm, 500–550 nm and 425–475 nm. The best fluorescence from the irradiated PRESAGE rods, compared with non-irradiated control rods, was achieved using the 638 nm laser and the 662–737 nm detector. This is the combination of laser and detector used for this study.

For consistency, settings such as pinhole, laser power and filters were fixed. The focal depth was also fixed at 2.5 mm, since shallower depths led to distortions owing to the curved surface of the rods. The gain on the photomultiplier tube (PMT) is probably the most important parameter for the fluorescent response, as it determines the fluorescence at which the PMT saturates, and therefore the useful range of the PMT. The PMT gain was selected and fixed at a value which gave a curve for 0–15 Gy fluorescent response that did not rise above 50% of the saturation level of the PMT. According to manufacturer’s specifications, the laser diode power stability is ensured to be constant to within 2%. All images taken were an average of eight scans to reduce image noise, and all seven valleys were sampled for the determination of PVDRs.

The images were saved as jp2000 files and examined using the *NIS-Element AR* software (version 3.10). The peak and valley regions were sampled, with the mean fluorescence value of 16 pixels (20 µm sample width) giving a reading of fluorescence, as a 12-bit number (0–4095). With the exception of experiment 5 (described in §3.5), all data were collected using a 10× objective lens and recorded as 512 × 512 images, giving a pixel size of 1.25 µm.

### 2.5. Flatbed scanning for GAF films   

**Figure 3:**
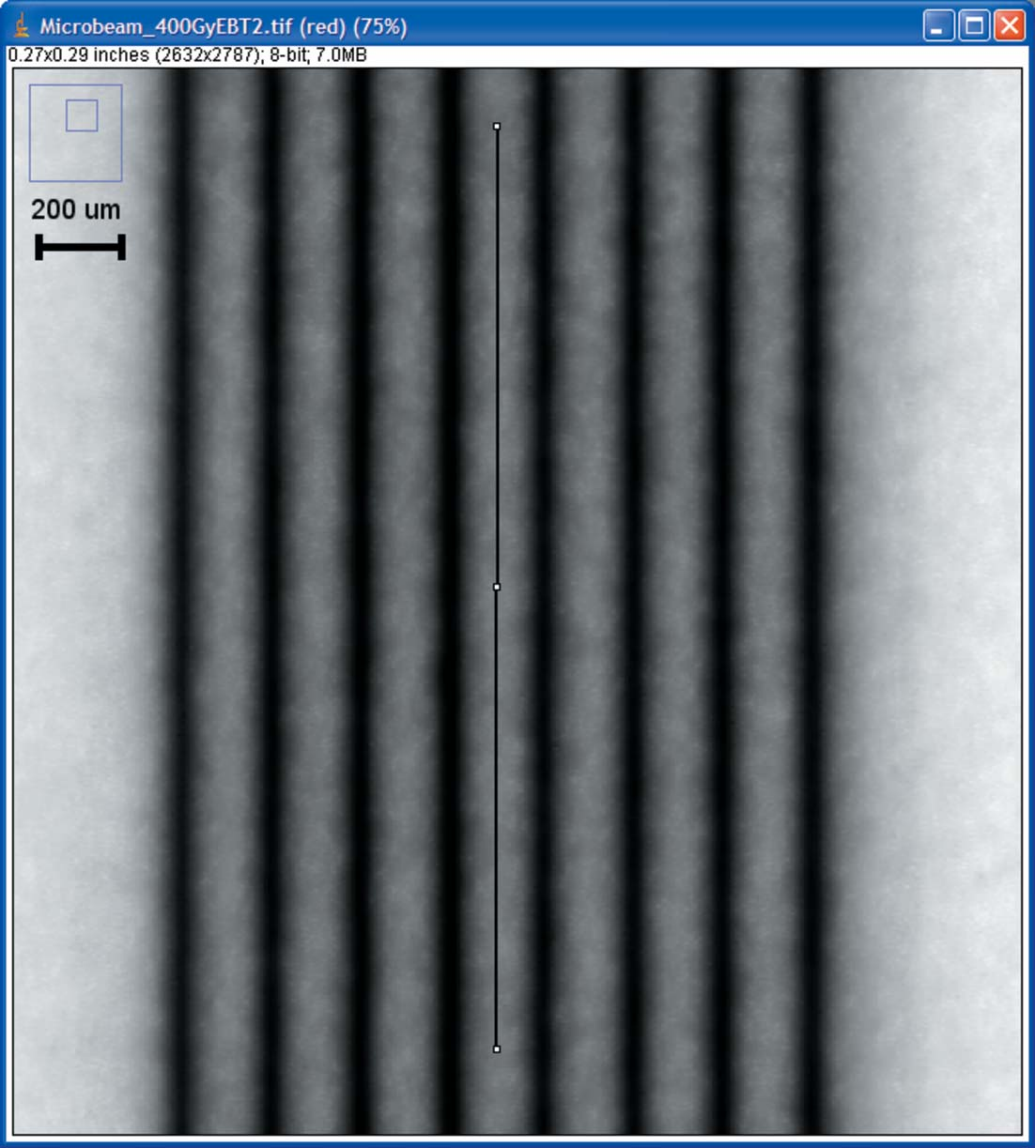
*ImageJ* screen capture of the analysis of a valley region of the EBT2 red channel response.

The GAFchromic film was analysed using an Epson V700 Professional scanner running *Epson Scan* (version 2.80E), at a resolution of 9600 dots per inch. The resulting image was saved as a TIF file. The three colour channels were split, and only the red channel data were analysed owing to its cleaner response than the blue or green channels, as recommended in EBT2 film specifications and other published data (Nariyama *et al.*, 2009 ▶; Hartmann *et al.*, 2010 ▶). The images taken were saved as compressionless TIF files and examined using *ImageJ* (version 1.37). The peak and valley regions were sampled as shown in Fig. 3 ▶ with a sampling width of 20 µm from the red channel response value giving a reading of optical density (OD), as an 8-bit number (0–255), for both peaks and valleys. All seven valleys were sampled for the determination of PVDRs. Data were then exported to *Minitab* (version 15) for analysis.

### 2.6. Calibration curve fitting by regression analysis   

Calibration response curves were developed by scanning microbeam-irradiated PRESAGE rods with peak doses between 0 and 15 Gy, and EBT2 films with peak doses between 2.5 and 75 Gy. A polynomial was fitted to the PRESAGE response, and a logarithmic response was fitted to the EBT2 response by regression analysis using Microsoft *Excel 2003*. This was done to coincide with the range of valley doses that would be measured. Subsequently, the valley regions of higher-dose irradiations were compared with these curves, and doses were interpolated for the valley regions of the microbeam when a known dose was delivered to the peak region. From this interpolated data the peak-to-valley ratio was calculated for individual valleys, and compared between the different dosimeters. This polynomial curve-fitting procedure is derived from Crosbie *et al.* (2008 ▶).

### 2.7. Statistical analysis   

All student’s *t*-tests mentioned in this paper were performed using *Minitab* (version 15), with a threshold of *p* = 0.05 used for establishing significance. Equal variances were not assumed.

## 3. Experimental outline   

The following experiments will be described in this paper, using the methods and materials outlined in §2.

### 3.1. Experiment 1: evaluating the PVDR using EBT2 film dosimetry   

GAFchromic EBT2 film is irradiated by an array of microbeams to various known peak doses. The measured OD was used to create a calibration curve against the delivered dose. Further EBT2 film is irradiation to peak doses that are far above the expected saturation dose that the film is capable of measuring to bring the valley dose up to the sensitive range. This value is compared against the calibration curve to determine the delivered valley dose and thus the PVDR. The PVDR is estimated using the formula
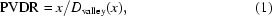
where *x* is the dose to the peak (in Gy), and *D*_valley_(*x*) is the corresponding dose delivered to the valley when the peak dose is *x*. The dose is evaluated as a function of the OD of light through the GAFchromic film; this function will be empirically determined. The EBT2 film peak OD responses from 2.5 Gy to 75 Gy was measured to establish the OD calibration curve, and the valleys from peak doses of 300 Gy to 800 Gy were then measured to estimate the PVDR. Although the calibration range of 2.5–75 Gy exceeds the specified dose range of the films of 40 Gy (Nariyama *et al.*, 2009 ▶), an accurate logarithmic approximation was still produced over this range.

### 3.2. Experiment 2: evaluating the PVDR using PRESAGE dosimetry   

Using the same mathematical approach outlined above for experiment 1, PRESAGE rods were irradiated by an array of microbeams to various known peak doses. The measured fluorescence was used to create a calibration curve against the delivered dose. Further rods were irradiation to peak doses that are far above the expected saturation dose that PRESAGE is capable of measuring to bring the valley dose up to the sensitive range. This value is compared against the calibration curve to determine the delivered valley dose and thus the PVDR. The PRESAGE peak fluorescent responses from 0 to 15 Gy were used to establish the PRESAGE fluorescence calibration curve, and the valleys from peak doses of 50 Gy to 150 Gy were then measured to estimate the PVDR.

### 3.3. Experiment 3: dose diffusion comparison between PRESAGE and EBT2 film   

In order to evaluate the comparative dose diffusion of PRESAGE and EBT2, 75 Gy-irradiated samples of each were compared and the full width at half-maximum (FWHM) was calculated for each dosimeter.

### 3.4. Experiment 4: using PRESAGE to measure the microbeam-collimated radiation’s diffusion through depth   

In order to demonstrate the ability of PRESAGE to record the lack of beam diffusion through depth from synchrotron-generated microbeams, a rod of PRESAGE was placed horizontally, and the microbeams shot lengthwise to a peak dose of 250 Gy. The irradiated PRESAGE was then scanned with the CLSM at depths of 20 mm and 100 mm, with the beamwidth compared between the two to verify beam spatial integrity. Three images were collected from each depth, with four peaks shown in each image. The FWHM values for the 12 peaks at each depth (20 mm and 100 mm) were then compared to see if there was any difference in beam width.

### 3.5. Experiment 5: determining the maximum resolution possible for measuring PRESAGE response by the CLSM system   

In order to demonstrate the upper limits of resolution of the CLSM, the ‘Maximum Resolution’ data were collected using the 40× objective lens and recorded as a 4096 × 4096 image, giving a nominal pixel size of 78 nm.

## 4. Results   

This section will present the results of the five experiments outlined in §3, one at a time.

### 4.1. Results from experiment 1: EBT2 films PVDR   

**Figure 4:**
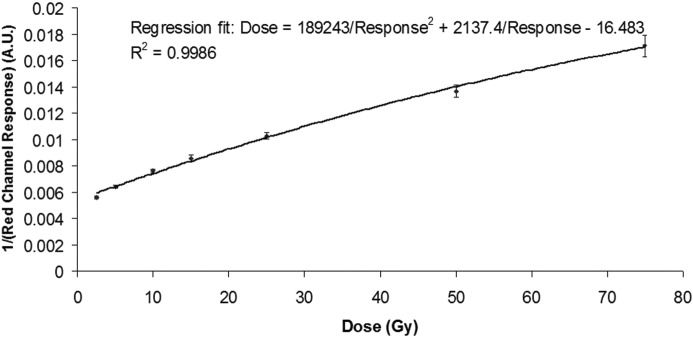
Calibration curve, by regression analysis of EBT2 film. Error bars denote the standard deviation.

**Figure 5:**
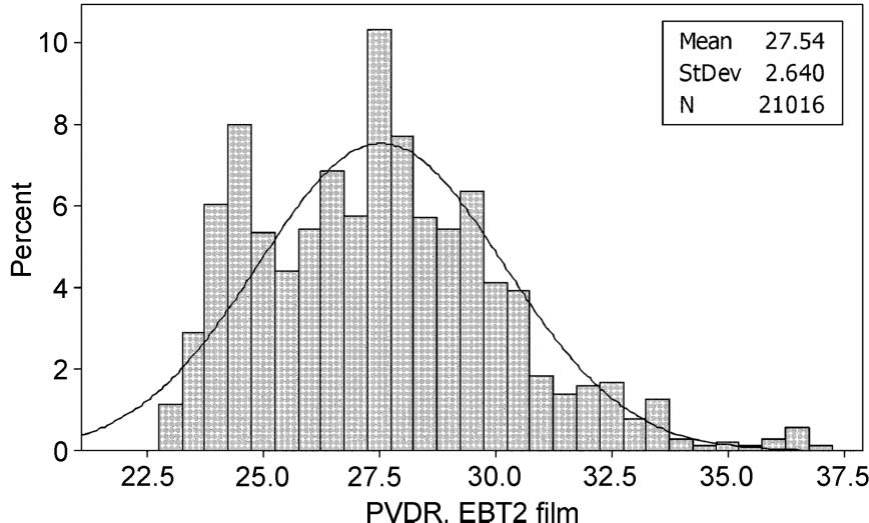
PVDR values from analysis of the EBT2 film scanned data.

The EBT2 film response to 2.5–75 Gy microbeams was analysed, and the regression analysis gave an approximation to
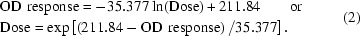
The valley response was sampled from over 4000 pixels from each EBT2 film that had been irradiated using the microbeam collimator to 300, 400, 600 and 800 Gy in the peak region. The range of valley doses from these films was measured to be between 10 and 35 Gy, which is within the range characterized by the EBT2 technical specifications (Nariyama *et al.*, 2009 ▶). After smoothing, each response value was then compared with the established response curve (Fig. 4 ▶) and the PVDR calculated, yielding a [mean ± standard deviation (s.d.)] PVDR value of 30.5 ± 2.6, as shown in Fig. 5 ▶.

### 4.2. Results from experiment 2: PRESAGE PVDR   

**Figure 6:**
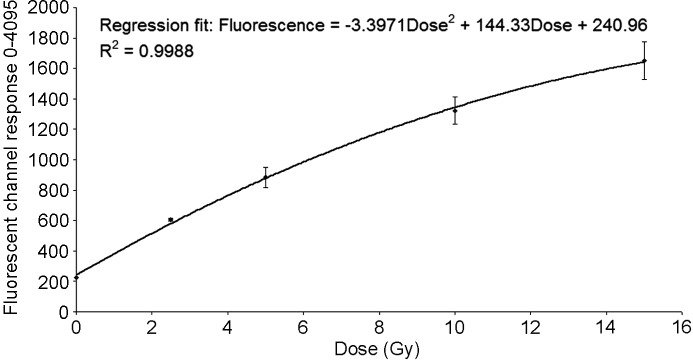
Calibration curve, by regression analysis of PRESAGE dosimeters. Error bars denote the standard deviation.

Using Microsoft *Excel 2003*’s polynomial fitting feature, a line of best fit was found for the PRESAGE response over the dose range 0–15 Gy. The data were modelled on a second-order polynomial as shown in Fig. 6 ▶. This equation was used to interpolate the valley doses from more heavily irradiated PRESAGE rods, using the quadratic formula to find the values of dose which satisfy the regression analysis equation when *ax* ^2^ + *bx* + *c* = 0, where *x* in this case is the dose, and *a*, *b* and *c* are −3.3971, 144.33 and (240.96 − fluorescence), respectively.

**Figure 7:**
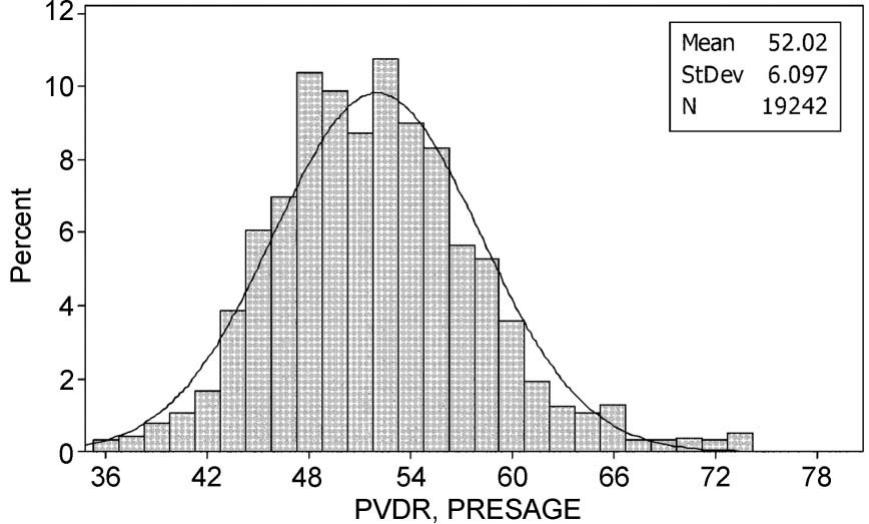
PVDR values obtained from PRESAGE fluorescence (measuring dose at a depth of 2.5 mm).

The valley fluorescence was sampled from over 4000 pixels from each PRESAGE dosimeter that had been irradiated to 50, 75, 100 and 150 Gy in the peak region. After smoothing, each fluorescence value was then compared with the established response curve and the PVDR calculated, yielding a (mean ± s.d.) PVDR value of 52.0 ± 6.1, as shown in Fig. 7 ▶.

### 4.3. Results from experiment 3: dose diffusion in EBT2 and PRESAGE   

**Figure 8:**
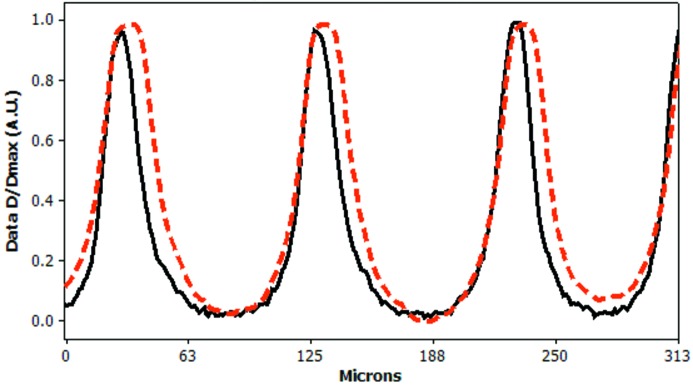
PRESAGE fluorescence (full line) and the transmitted light from GAFchromic film (dashed line), normalized to their own internal maximum and minimum values. PRESAGE fluorescence returns a narrower tighter beam width than the GAFchromic film, indicating less diffusion. These were taken from images captured at 512 × 512 pixel resolution with a 10× objective magnification.

At a 10× objective magnification, the transmission of light through a 75 Gy microbeam-irradiated GAFchromic EBT2 was compared with the fluorescence of a similarly irradiated PRESAGE rod. The resultant plot in Fig. 8 ▶ shows that the PRESAGE fluorescence measurements exhibit much less diffusion than the EBT2 film, returning a narrower peak. The FWHM values for six PRESAGE fluorescence peaks average (mean ± s.d.) 25.6 ± 1.5 µm, and six EBT2 film peaks average (mean ± s.d.) 39.8 ± 2.7 µm, giving a significant difference on an unpaired *t*-test (*p* < 0.01).

### 4.4. Results from experiment 4: beam diffusion with depth   

**Figure 9:**
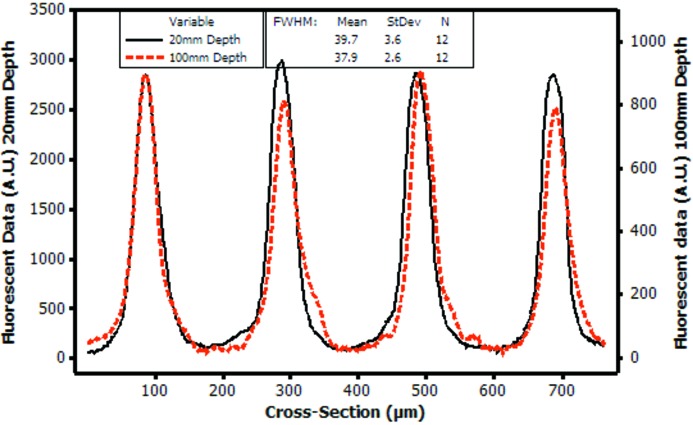
Microbeam profile showing minimal diffusion of the microbeam at a depth of 20 mm (full line) and 100 mm (dashed line) of the PRESAGE dosimeter.

Fig. 9 ▶ compares the fluorescent images captured by the CLSM of microbeams at a depth of 20 mm and 100 mm through the rod. The FWHM of the beam profile was calculated for the 12 peaks sampled at each depth, giving a FWHM of (mean ± s.d.) 39.7 ± 3.6 µm at 20 mm depth, and 37.9 ± 2.6 µm at 100 mm depth. This did not show a significant difference on a two-sample *t*-test (*p* = 0.161), and confirms that the microbeam collimation is not diffusing through to a clinically useful depth. Note that the FWHM measurements obtained in this experiment are much higher than for the PRESAGE rod used in experiment 3, since the peak dose was 250 Gy (compared with 75 Gy in experiment 3). It is therefore expected that the collimated beam’s penumbra extends to create a wider FWHM reading.

### 4.5. Results from experiment 5: microscope settings for maximum resolution   

**Figure 10:**
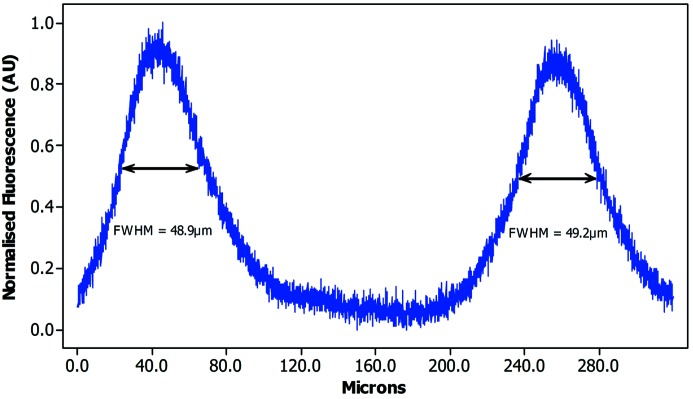
Maximum resolution of the PRESAGE dosimeter, at 40× objective magnification. This represents the best possible pixel density, showing a FWHM measurement for each peak of 49.2 µm and 48.9 µm.

The maximum resolution recordable by the CLSM is a 4096 × 4096 pixel image, and the maximum objective magnification used was 40×. This yields a pixel resolution, in the *XY* plane, of 78 nm. Fig. 10 ▶ shows the result of such a scan of a PRESAGE rod, showing a FWHM measurement of 49 µm, compared with the 20.5 µm measured at 10× magnification. This illustrates the importance of fixed microscope settings when determining beam characteristics and comparing results from different samples using the same settings, and shows the upper limits of spatial resolution of the detection system.

## 5. Discussion   

This work shows the first known application of fluorescent microscopy to a PRESAGE dosimeter, and demonstrates the effectiveness of this technique to measure the synchrotron-generated microbeam’s important peak-to-valley dose ratio. This paper also shows that PRESAGE fluorescence can illustrate the well known property of microbeam-collimated synchrotron beams to maintain collimation through to a clinically useful depth. Normal broad-beam radiotherapy (where field sizes are measured in centimetres) has no need for pixel resolutions as high as those achieved here, but the high dose gradient inherent to microbeam collimation demands that the measurement technique be accurate down to the micrometre scale.

It should be noted that the large standard deviations of the PVDR measurements should not come as a surprise, since the valley doses at the centre of a microbeam array is inherently higher, owing to scatter from more adjacent peaks, than valley doses at the edge of a microbeam array (De Felici *et al.*, 2005 ▶). Since all seven valley doses were used to establish the PVDRs in this study, this variation is expected.

The two different PVDRs calculated using EBT2 film and PRESAGE (30.5:1 and 52:1, respectively) clearly need explanation. It is thought that the main reason for this disagreement is illustrated in Fig. 8 ▶, where the dose response of PRESAGE is shown to have a much narrower response curve, illustrating that dose diffusion through EBT2 films may be limiting its usefulness to microbeam valley measurements. This is thought to be due to the higher sensitivity to low doses of the EBT2 compared with the PRESAGE rods. A lower PVDR measured by film was also noticed by Torikoshi *et al.* (2008 ▶), who concluded that scattering owing to silver within the film was responsible for their higher than expected valley dose; however, this cannot be the case with the EBT2 films used in this study which uses no high-*Z* materials.

It is well established that measuring the PVDR with different methods, different dosimeters and at different distances from the collimator will yield different results. This can be seen in the results summarized by Crosbie *et al.* (2008 ▶), where the PVDR is estimated at a depth of less than 1 cm as varying between 29:1 and 53:1. To compound this uncertainty, there is no single definition of how wide the valley region should be considered to be, or at what depth or distance from the collimator it should be measured at (Haryanto *et al.*, 2004 ▶; Siegbahn *et al.*, 2009 ▶). The EBT2 PVDR figure of 30.5:1 was taken from surface measurements, whereas the PRESAGE PVDR of 52.0:1 was taken from a depth of 2.5 mm. It was necessary to read the PRESAGE dosimeters at this depth owing to the curved surface of the dosimeter. This difference in PVDR value is in line with other papers that have found that PVDR measurements increase after a few millimetres depth (De Felici *et al.*, 2005 ▶; Crosbie *et al.*, 2008 ▶; Wong, 2009 ▶). These values are also within the range of other calculated PVDRs published for similar geometries and spectra, as summarized in Table 1 ▶.

It is also worth noting that the PVDR values calculated in this paper are independent of the true dose rate of the beamline, which as previously stated has been assumed for the sake of calculation to be 120 Gy s^−1^ (Nariyama *et al.*, 2009 ▶). Since the beamline dose is actually determined by the total shutter open time of the beamline, the expressions of dose (in Gy) found in this paper are really scalar multiples of beam exposure time. Since this beam exposure time was used for the curve fitting/calibration calculations, and also for the selection of peak dose for the measurement of valley dosimeter response, it would not affect the final analysis of the PVDRs if the true dose rate of the beamline were very different from the assumed value of 120 Gy s^−1^. Also, a slightly different set of CLSM settings would produce a different calibration curve and valley fluorescent response from the PRESAGE rods (as demonstrated by the change in FWHM in Figs. 8 ▶ and 9 ▶), but that would not be expected to alter the calculated PVDR value.

**Figure 11:**
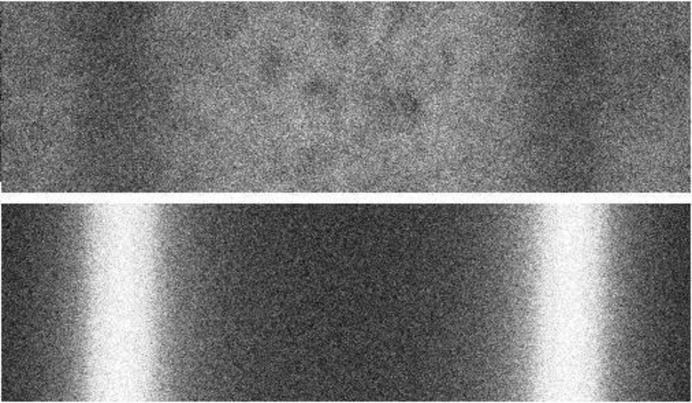
Fluorescent channels of EBT2 film (top) and PRESAGE (bottom). Note that fluorescence was still detected on the film, but not as clearly as on the PRESAGE dosimeter.

It was also found that the GAFchromic films (which are designed to be read by light transmission) also fluoresce with the use of a 638 nm laser, although not as intensely as the PRESAGE dosimeter (Fig. 11 ▶). These data were not used in the subsequent analysis; however, it is noted here to demonstrate that laser-induced fluorescence is not unique to PRESAGE. Also note that the inhomogeneities present in the EBT2 film response are indicative of the inherent limitations to resolution as discussed by Soares (2006 ▶), which is thought to be due to an uneven distribution of chromophores within the emulsion. Another variable worth noting is the one-month storage time before analysis of the PRESAGE rods. Although radiochromic response is known to slightly diminish over time in PRESAGE dosimeters (Adamovics & Maryanski, 2006 ▶), this will affect both the peak and valley regions and so is not thought to be a limiting factor to this study.

From a clinical perspective the ratio of irradiated area to unirradiated area is of great significance, since it will determine the post-treatment recovery of the tissue. As noted in experiment 5, changing the CLSM gain and magnification settings does alter the apparent width of the beam. This effect must be further investigated before this methodology is clinically used.

## 6. Conclusion   

The dose record contained in PRESAGE dosimeters can be read at high resolution by the fluorescent channels of a confocal microscope. It has also shown that the dose recorded in a PRESAGE gel is capable of much better resolution than is used by current OCT systems. PRESAGE is capable of recording dose deposition with very high resolution, owing to its low ion diffusion. By contrast, the optical density and fluorescence data collected from GAFchromic films shows significant ion diffusion, rendering it unsuitable for recording dose profiles at submicrometre resolution.

Fluorescent microscopy provides a novel methodology of obtaining dose distribution data from PRESAGE rods at submicrometre resolution. Using this, we have shown that microbeams do not diffuse through 100 mm of tissue-equivalent material, and that this approach has sufficient spatial resolution to analyze the peak-to-valley ratio of microbeams. For future work, a depth–dose profile and a study of PVDR through depth will be generated using this methodology with stacks of flat-sided PRESAGE dosimeters. It is also feasible to convert such fluorescent data into a tomogram, in order to create a full three-dimensional rendering of the dose profile contained in a PRESAGE rod.

## Figures and Tables

**Table 1:** PVDR values from similar studies

Author	PVDR	Depth	Measurement method	Spectrum
Slatkin *et al.* (1992 ▶)	35	01cm	CPE Monte Carlo	50, 100 and 150keV monochromatic X-rays
Crosbie *et al.* (2008 ▶)	55	0	EBT and HD810 film	Mean photon energy 125keV
Siegbahn *et al.* (2006 ▶)	29	01 cm	Penelope Monte Carlo	Mean photon energy 107keV
De Felici *et al.* (2005 ▶)	40	0.5 cm	EGS4 Monte Carlo	Peak photon fluence at 107keV
Nariyama *et al.* (2009 ▶)	77	0.1 cm	HD810 film	Peak photon fluence at 90keV
Torikoshi *et al.* (2008 ▶)	1217	0	EDR2 film	Peak photon fluence at 90keV
Annabell (this work)	31	0	EBT2 film	Peak photon fluence at 90keV
Annabell (this work)	52	0.25 cm	PRESEAGE fluorescence	Peak photon fluence at 90keV

## References

[bb1] Adamovics, J. & Maryanski, M. J. (2006). *Radiat. Prot. Dosimetry*, **120**, 107–112. 10.1093/rpd/nci555 16782984

[bb2] Clift, C., Thomas, A., Adamovics, J., Chang, Z., Das, I. & Oldham, M. (2010). *Phys. Med. Biol.* **55**, 1279–1293. 10.1088/0031-9155/55/5/002 PMC3030986 20134082

[bb3] Crosbie, J. C., Svalbe, I., Midgley, S. M., Yagi, N., Rogers, P. A. W. & Lewis, R. A. (2008). *Phys. Med. Biol.* **53**, 6861–6877. 10.1088/0031-9155/53/23/014 19001701

[bb4] De Felici, M., Felici, R., Sanchez del Rio, M., Ferrero, C., Bacarian, T. & Dilmanian, F. A. (2005). *Med. Phys.* **32**, 2455–2463. 10.1118/1.1951043 16193774

[bb5] Dilmanian, F. A., Qu, Y., Feinendegen, L. E., Peña, L. A., Bacarian, T., Henn, F. A., Kalef-Ezra, J., Liu, S., Zhong, Z. & McDonald, J. W. (2007). *Exp. Hematol.* **35**(4, Suppl.), 69–77. 10.1016/j.exphem.2007.01.014 17379090

[bb6] Doran, S. J., Brochard, T., Adamovics, J., Krstajic, N. & Bräuer-Krisch, E. (2010). *Phys. Med. Biol.* **55**, 1531–1547. 10.1088/0031-9155/55/5/018 20157228

[bb7] Guo, P., Adamovics, J. & Oldham, M. (2006). *Med. Phys.* **33**, 3962–3972. 10.1118/1.2349686 PMC1780266 17089858

[bb8] Hartmann, B., Martisikova, M. & Jakel, O. (2010). *Med. Phys.* **37**, 1753–1756. 10.1118/1.3368601 20443496

[bb9] Haryanto, F., Fippel, M. & Bakai, A. (2004). *Strahlenther. Onkol.* **180**, 57–61. 10.1007/s00066-004-1135-3 14704846

[bb10] Martinez-Rovira, I., Sempau, J., Fernández-Varea, J. M., Bravin, A. & Prezado, Y. (2010). *Phys. Med. Biol.* **55**, 4375–4388. 10.1088/0031-9155/55/15/012 20647606

[bb11] Nariyama, N., Ohigashi, T., Umetani, K., Shinohara, K., Tanaka, H., Maruhashi, A., Kashino, G., Kurihara, A., Kondob, T., Fukumoto, M. & Ono, K. (2009). *Appl. Radiat. Isotop.* **67**, 155–159. 10.1016/j.apradiso.2008.08.002 18789708

[bb12] Nettelbeck, H., Takacs, G. J., Lerch, M. L. F. & Rosenfeld, A. B. (2009). *Med. Phys.* **36**, 447–456. 10.1118/1.3049786 19291983

[bb13] Rahman, A. T. A., Bräuer-Krisch, E., Brochard, T., Adamovics, J., Bradley, D. & Doran, S. (2010). *J. Phys. Conf. Ser.* **250**, 012083.

[bb14] Sakhalkar, H. S., Adamovics, J., Ibbott, G. & Oldham, M. (2009). *Med. Phys.* **36**, 71–82. 10.1118/1.3005609 PMC2673667 19235375

[bb15] Siegbahn, E. A., Bräuer-Krisch, E., Bravin, A., Nettelbeck, H., Lerch, M. L. F. & Rosenfeld, A. B. (2009). *Med. Phys.* **36**, 1128–1137. 10.1118/1.3081934 19472618

[bb16] Siegbahn, E. A., Stepanek, J., Bräuer-Krisch, E. & Bravin, A. (2006). *Med. Phys.* **33**, 3248–3259. 10.1118/1.2229422 17022219

[bb17] Slatkin, D. N., Dilmanian, F. A., Spanne, P. & Sandborg, M. (1992). *Med. Phys.* **19**, 1395–1400. 10.1118/1.596771 1461201

[bb18] Soares, C. G. (2006). *Radiat. Meas.* **41**, S100–S116.

[bb19] Spiga, J., Siegbahn, E. A., Bräuer-Krisch, E., Randaccio, P. & Bravin, A. (2007). *Med. Phys.* **34**, 4322–4330. 10.1118/1.2794170 18072497

[bb20] Torikoshi, M., Ohno, Y., Yagi, N., Umetani, K. & Furusawa, Y. (2008). *Eur. J. Radiol.* **68**(3, Suppl.), S114–S117. 10.1016/j.ejrad.2008.04.052 18602783

[bb21] Wong, C. J. (2009). PhD thesis, RMIT University, Melbourne, Australia.

[bb22] Yukihara, E. G., Guduru, S., Mardirossian, G., Mirzasadeghi, M. & Ahmad, S. (2008). *Med. Phys.* **35**, 260–269. 10.1118/1.2816106 18293581

